# Supplementary material for: Cyclophilin Inhibitor Rencofilstat Combined with Proteasome Inhibitor Ixazomib Increases Proteotoxic Cell Death in Advanced Prostate Cancer Cells with Minimal Effects on Non-Cancer Cells
Source: Biomedicines. 2025 Oct 7;13(10):2442. doi: 10.3390/biomedicines13102442 (PMC12561735; doi:10.3390/biomedicines13102442)
Supplement: Supplementary file 1 [file biomedicines-13-02442-s001.zip › Supplementary.pdf]

Supplementary Tables 1, 2
Supplementary Figures 1-12

Cyclophilin Inhibitor Rencofilstat Combined with Proteasome Inhibitor Ixazomib Increases Proteotoxic Cell Death in Advanced Prostate Cancer Cells with Minimal Effects on Non-Cancer Cells

Carlos Perez-Stable, Alicia de las Pozas, Medhi Wangpaichitr Robert Foster, Daren Ure

Figure S1

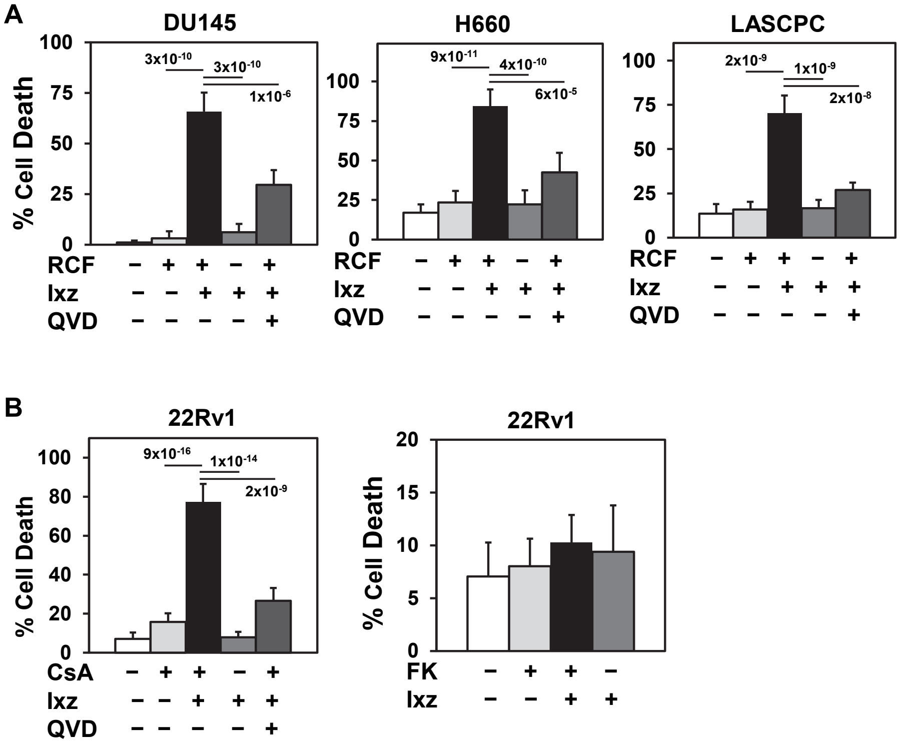

Figure S1. RCF or CsA + Ixz combination enhances apoptotic cell death in CRPC/NEPC cells. **(A)** Trypan blue exclusion assay (72 h) showed significantly higher cell death in RCF (10 μM DU145; 5 μM H660/LASCPC) + Ixz (9708, prodrug form; 50 nM DU145, 15 nM H660, 25 nM LASCPC) treated DU145, H660, LASCPC compared to RCF, Ixz, and control treated cells. Addition of apoptosis inhibitor QVD (10 μM) significantly decreased RCF + Ixz cell death. **(B)** Trypan blue exclusion assay showed significantly higher cell death in CsA (10 μM) + Ixz (9708; 25 nM) treated 22Rv1 compared to CsA, Ixz, and control treated cells. Addition of apoptosis inhibitor QVD (10 μM) significantly decreased CsA + Ixz cell death. In contrast, FK506 (10 μM; non-Cyp binding immunosuppressor) + Ixz (25 nM) had no effect on cell death in 22Rv1. *p* values are shown near the bars.

Table S1

Table S1 RCF + Ixz synergistically inhibits LNCaP, 22Rv1, and PC3

	RCF (μM)	FA	Ixz (nM)	FA	FA (RCF+Ixz)	CI
LNCaP	10	0.38	25	0.25	0.76	0.45
	10	0.38	50	0.39	0.87	0.46
22Rv1	10	0.32	25	0.13	0.73	0.64
	10	0.32	50	0.19	0.84	0.51
PC3	10	0.29	25	0.11	0.94	0.38
	10	0.29	50	0.42	0.98	0.32

Cell proliferation assay showed that various combinations of RCF + Ixz (2238) synergistically inhibited LNCaP, 22Rv1, and PC3 as determined by combination index (CI). FA, fraction affected refers to inhibition (no inhibition control=0; 100% inhibition=1.0).

Table S2

Table S2 RCF or CsA + Ixz synergistically inhibits PCa, CRPC, and NEPC cells.

	RCF (μM)	FA	Ixz (nM)	FA	FA (RCF+Ixz)	CI
DU145	10	0.30	25	0.17	0.94	0.27
	10	0.30	50	0.33	0.95	0.34
H660	5	0.27	15	0.54	0.92	0.55
	5	0.27	20	0.64	0.97	0.39
LASC	10	0.51	25	0.19	0.92	0.29
	10	0.51	50	0.29	0.97	0.03

	CsA (μM)	FA	Ixz (nM)	FA	FA (CsA+Ixz)	CI
LNCaP	10	0.30	25	0	0.71	0.34
	10	0.30	50	0.02	0.69	0.42
22Rv1	10	0.37	25	0.04	0.60	0.49
	10	0.37	50	0.11	0.89	0.08
PC3	10	0.12	25	0	0.77	0.08
	10	0.12	50	0.17	0.93	0.11

Cell proliferation assay showed various combinations of RCF or CsA + Ixz (9708, prodrug form) synergistically inhibited PCa (LNCaP), CRPC (22Rv1, DU145, PC3), and NEPC (H660, LASCPC) as determined by combination index (CI). FA, fraction affected refers to inhibition (no inhibition control=0; 100% inhibition=1.0).

Figure S2

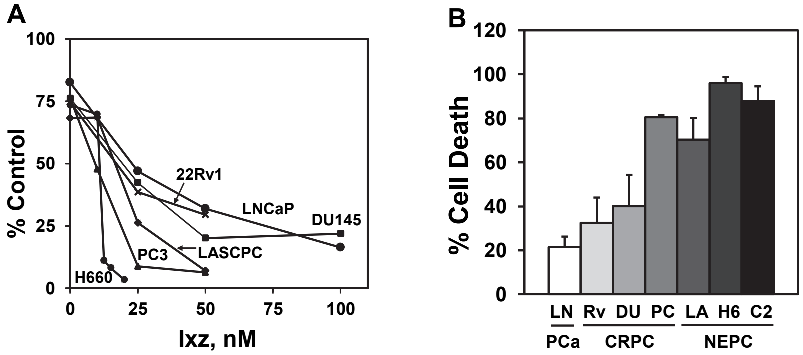

Figure S2. NEPC cells are more sensitive to RCF + Ixz. **(A)** Cell viability assay (72 h) showed the combinations of RCF (5 μM) + varying concentrations of Ixz (9708, prodrug form; 10-100 nM) in PCa (LNCaP), CRPC (22Rv1, PC3, DU145), and NEPC (H660, LASCPC) cells. The most sensitive cells were NEPC (H660, LASCPC) and CRPC (PC3). LNCaP PCa and 22Rv1, DU145 CRPC were the least sensitive. Values are % of control (=100%). **(B)** Trypan blue exclusion assay (72 h) showed RCF (5 μM) + Ixz (9708; 25 nM or 50 nM [DU145, PC3]) increased greater cell death in NEPC (H660, LASCPC, TRAMP-C2) and PC3 (70-96%) compared LNCaP, 22Rv1, and DU145 (21-40%). *n*=6-10, 3 independent experiments.

Figure S3

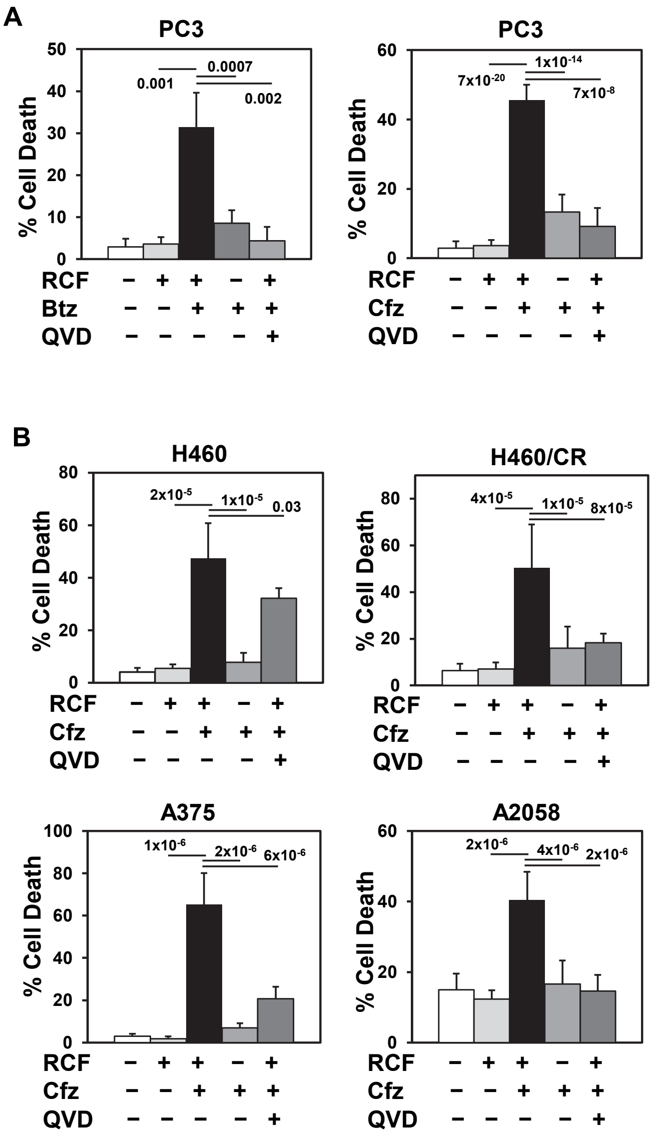

Figure S3. RCF + Btz or Cfz enhances apoptotic cell death in CRPC, non-small cell lung cancer, and melanoma. **(A)** Trypan blue exclusion assay (48 h) showed additional UPS inhibitors Btz (10 nM) and Cfz (25 nM) also significantly increased apoptotic cell death when combined with RCF (1 µM) in PC3 compared to RCF, Cfz, and control treated cells. **(B)** Trypan blue exclusion assay (48 h) showed significantly higher apoptotic cell death in RCF (1 µM) + Cfz (25 nM) (72h) treated H460, and H460/CR (Cfz 15 nM) non-small cell lung cancer compared to RCF, Ixz, and control treated cells. Similar results were obtained in A375 (Cfz 15 nM; 48 h) and A2058 (Cfz 20 nM; 48 h) melanoma cells. Early investigations used a low concentration of RCF before increasing doses for later experiments. *p* values are shown above the bars.

Figure S4

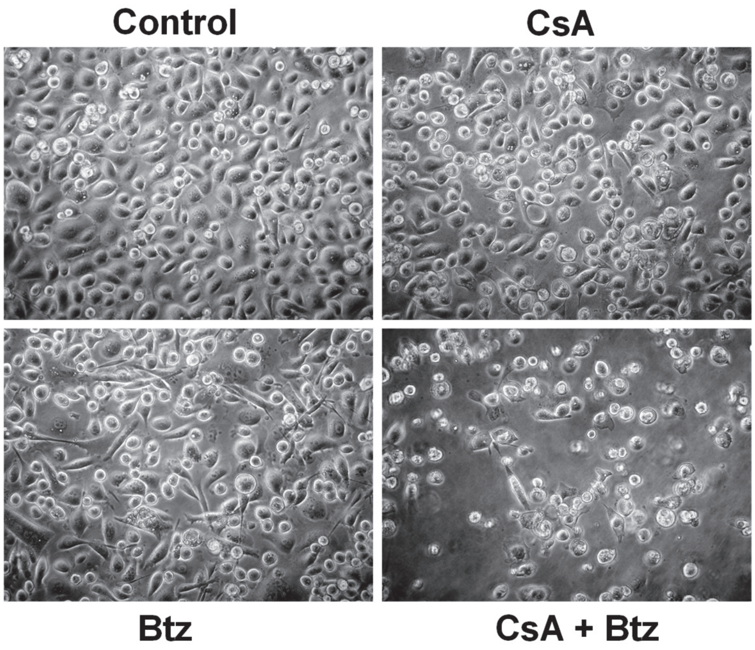

Figure S4. CsA + Btz has strong morphological effects on PC3 cells. Microscopic images (x100) of PC3 cells treated with CsA (10 μM) + Btz (10 nM) for 24 h resulted in significant changes in cell morphology compared to CsA, Btz, and control cells.

Figure S5

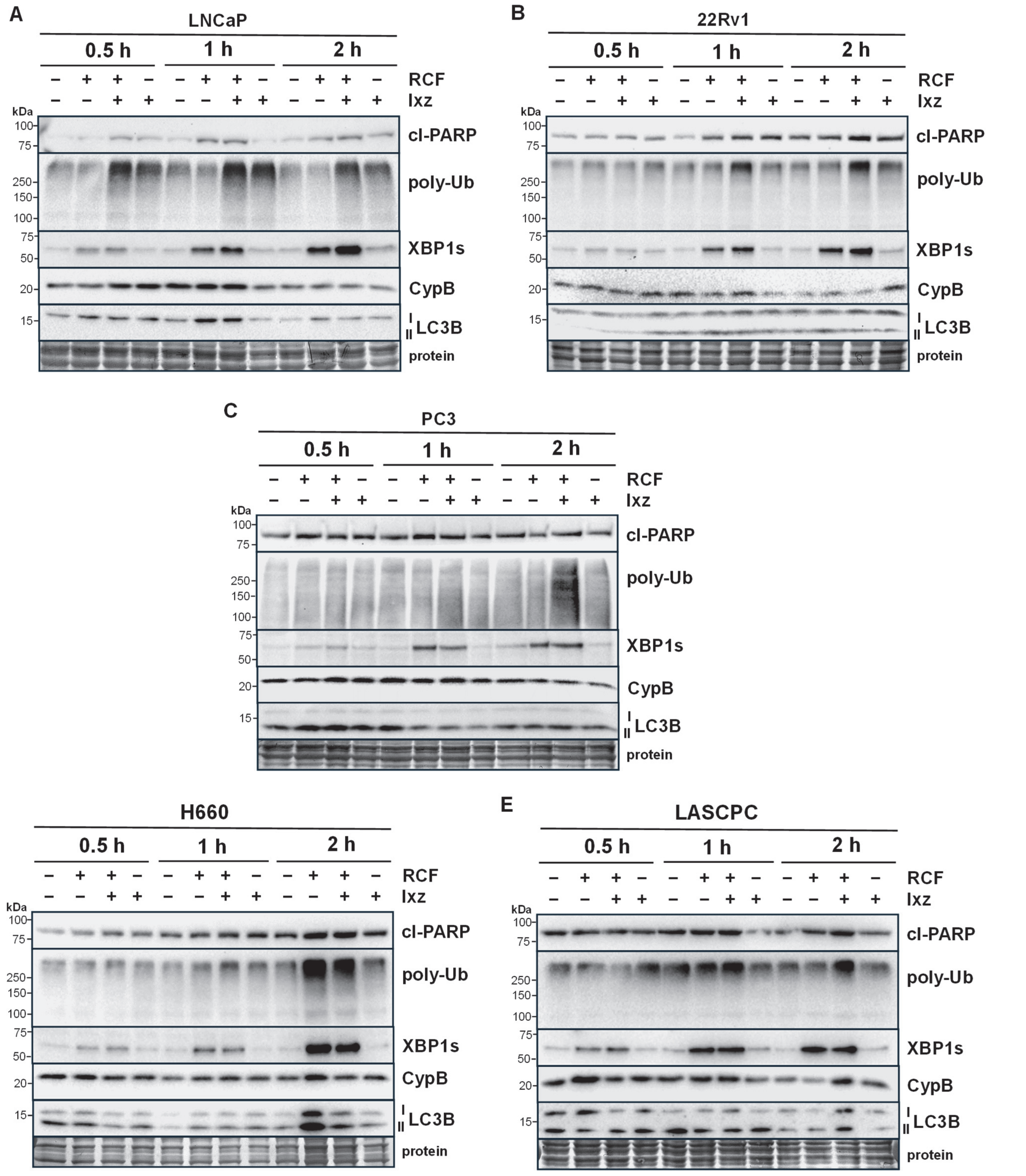

Figure S5. Early increase in XBP1s in RCF alone and RCF + Ixz treated PCa/CRPC/NEPC cells. Western blot analysis showed early increased XBP1s in RCF (10 μM) alone and RCF + Ixz (2238, active form; 50 nM) treated (**A)** LNCaP, **(B)** 22Rv1 (Ixz 25 nM), and **(C)** PC3 (Ixz 25 nM) (0.5–2h) compared to RCF, Ixz, and control treated cells. RCF + Ixz also increased poly-Ub at an early time (0.5–2 h). No clear differences were noted for cl-PARP (except for slight increase in LNCaP), CypA/B, and LC3B. Similar results were obtained in **(D)** H660 and **(E)** LASCPC NEPC cells using RCF (5 μM) + Ixz (25 nM). Size of molecular weight markers in kDa shown to the left. Protein refers to Coomassie blue stain after all immunological analysis was completed.

Figure S6

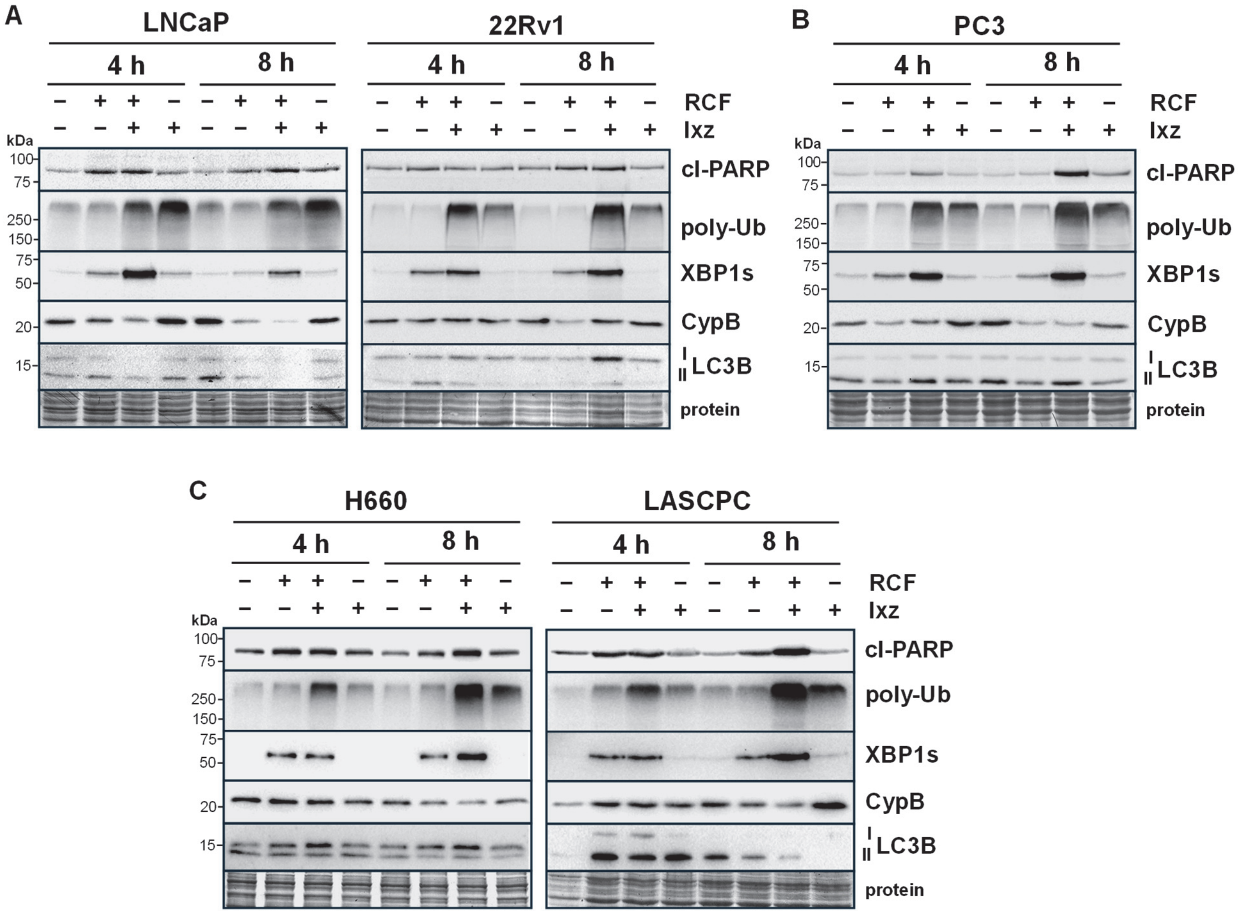

Figure S6. XBP1s and poly-Ub are maintained at higher levels in RCF + Ixz. Western blot analysis showed at later times (4, 8 h), RCF (10 µM) + Ixz (2238, active form) maintained high XBP1s and poly-Ub (also increased by Ixz alone) in **(A)** LNCaP (Ixz 50 nM), 22Rv1 (Ixz 25 nM), **(B)** PC3 (Ixz 25 nM), and **(C)** H660, LASCPC (RCF 5 µM + Ixz 25 nM). There was a slight increase in cl-PARP and decreased CypB (4, 8 h). No clear differences were noted with LC3B. Size of molecular weight markers in kDa shown to the left. Protein refers to Coomassie blue stain after all immunological analysis was completed.

Figure S7

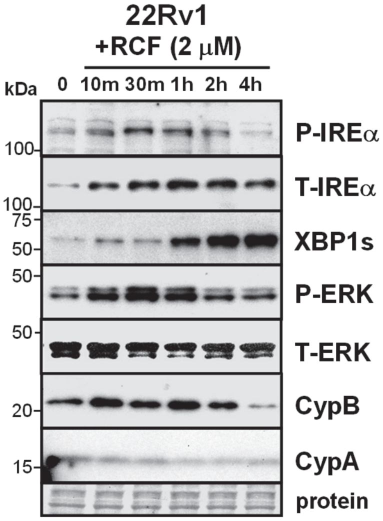

Figure S7. Immediate early effects of RCF alone in 22Rv1 CRPC cells. Western blot analysis showed an immediate early increase in (P/T)-IRE1α and P-ERK (10, 30 min [m]) followed by XBP1s (1 h) and decreased CypB (4 h) in RCF (2 μM) treated 22Rv1. No clear differences in CypA were noted. Size of molecular weight markers in kDa shown to the left. Protein refers to Coomassie blue stain after all immunological analysis was completed.

Figure S8

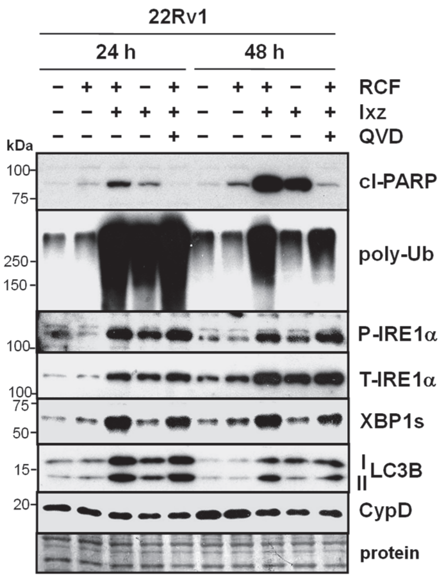

Figure S8. Expected differences in cl-PARP, poly-Ub, P/T-IRE1α, XBP1s, LC3B, and CypD in LNCaP treated with RCF (2 μM) + Ixz (9708, prodrug form; 100 nM) for 24 h (same protein lysates used in Figure 2 [−TG]) (Western blot). Size of molecular weight markers in kDa shown to the left. Protein refers to Coomassie blue stain after analysis was completed.

Figure S9

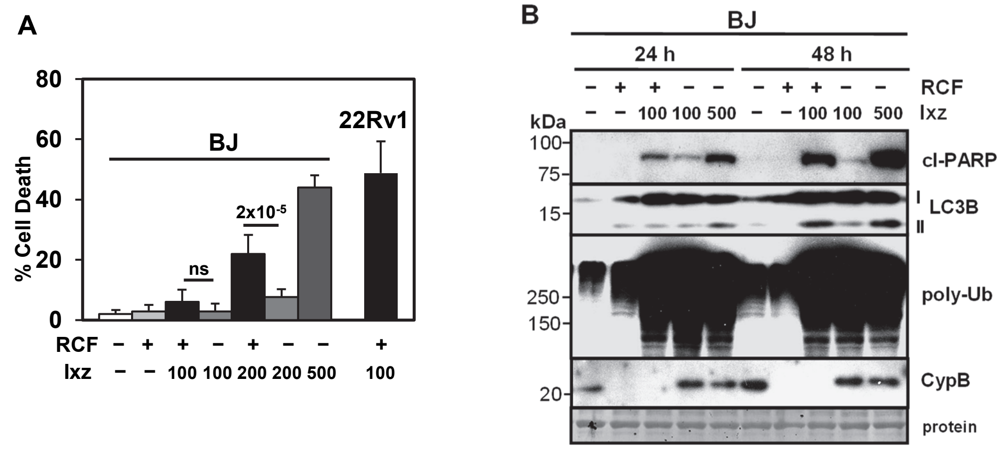

Figure S9. RCF + Ixz has less effect on cell death in BJ non-cancer cells. **(A)** Trypan blue exclusion assay (72 h) showed RCF (2 μM) + Ixz (9708, prodrug form; 100 nM) did not significantly increase cell death compared to RCF, Ixz, and control treated BJ cells. In contrast, RCF + Ixz greatly increased cell death in 22Rv1 CRPC (49%). RCF + Ixz (200 nM) increased cell death in BJ, whereas Ixz 500 nM was toxic. *p* value shown above the bar; ns, not significant. **(B)** Western blot analysis (24, 48 h) showed RCF + Ixz increased cl-PARP, poly-Ub, LC3B and decreased CypB (also with RCF) in BJ cells. Ixz 500 nM is positive toxicity control, and protein refers to Coomassie blue stain after all immunological analysis was completed.

Figure S10

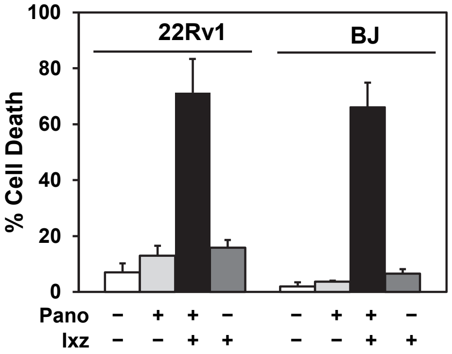

Figure S10. Combination of pan-HDAC inhibitor panobinostat + Ixz is toxic in both 22Rv1 CRPC and BJ non-cancer cells. Trypan blue exclusion assay (72 h) showed panobinostat (Pano; 10 nM) + Ixz (9708, prodrug form; 100 nM) significantly increased cell death in both BJ and 22Rv1 compared to Pano, Ixz, and control treated cells.

Figure S11

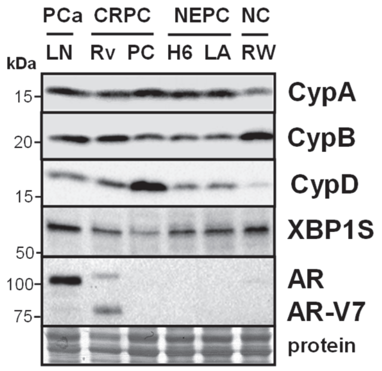

Figure S11. Comparison of Cyp and XBP1S protein levels in PCa/CRPC/NEPC and RWPE-1 non-cancer cells. Western blot analysis showed CypA is 2.8-fold lower in RWPE-1 (RW) non-cancer (NC) compared to LNCaP (LN) (=1) (PCa), 22Rv1 (Rv), PC3 (PC) (CRPC), H660 (H6), and LASCPC (LA) (NEPC). CypB was 2-fold lower in PC3/NEPC compared to LNCaP, 22Rv1, and RWPE-1. CypD was highest in PC3 and very low in RWPE-1. XBP1s was lowest in PC3 and similar in all other cells. Also shown was expression of AR and AR-V7. Size of molecular weight markers in kDa shown to the left. Protein refers to Coomassie blue stain after all immunological analysis was completed.

Figure S12

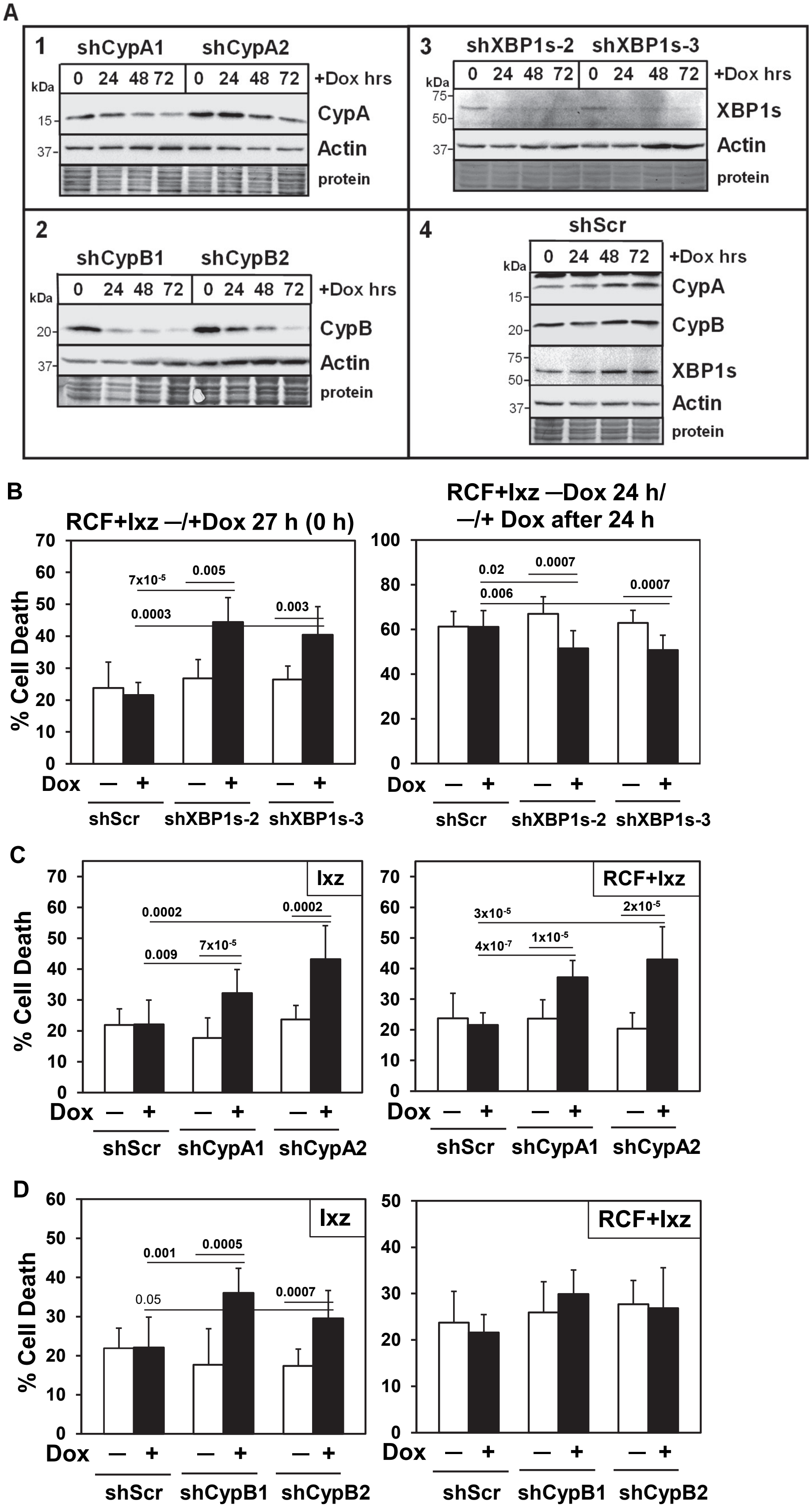

A
1
shCypA1
shCypA2
kDa
0 24 48 72 0 24 48 72
+Dox hrs
15
CypA
37
Actin
protein
3
shXBP1s-2
shXBP1s-3
0 24 48 72 0 24 48 72
+Dox hrs
75
50
XBP1s
37
Actin
protein
2
shCypB1
shCypB2
0 24 48 72 0 24 48 72
+Dox hrs
20
CypB
37
Actin
protein
4
shScr
0 24 48 72
+Dox hrs
CypA
CypB
XBP1s
Actin
protein
B
RCF+Ixz —/+Dox 27 h (0 h)
RCF+Ixz —Dox 24 h/ —/+ Dox after 24 h
% Cell Death
7x10-5
0.005
0.0003
0.003
0.02
0.0007
0.006
0.0007
Dox — + — + — +
shScr
shXBP1s-2
shXBP1s-3
C
Ixz
RCF+Ixz
0.0002
0.0002
0.009
7x10-5
3x10-5
2x10-5
4x10-7
1x10-5
shScr
shCypA1
shCypA2
D
Ixz
RCF+Ixz
0.001
0.0005
0.05
0.0007
shScr
shCypB1
shCypB2

Figure S12. Inducible knockdowns: XBP1s is pro-survival early but pro-cell death later whereas CypA and CypB are pro-survival in RCF + Ixz treated LNCaP PCa cells. **(A)** Western blot analysis of **(1)** LNCaP/shCypA-1, -2; **(2)** LNCaP/shCypB-1, -2; and **(3)** LNCaP/shXBP1s-2, -3 cells treated with Dox (100 ng/ml) for 24, 48, and 72 h. Results showed decreased CypA (1), CypB (2), and XBP1s (3) compared to control cells; no effect on actin was noted. Dox treatment of LNCaP/shScr negative control (4) showed no clear differences in CypA, B, XBP1s, or actin (same blot). Size of molecular weight markers in kDa shown to the left. Protein refers to Coomassie blue stain after all immunological analysis was completed. **(B)** Trypan blue exclusion assays showed addition of Dox (+) to induce knockdown of XBP1s at time 0 h increased cell death in RCF (10 μM) + Ixz (2238, active form; 50 nM) treated LNCaP/shXBP1s-2 and -3 (27 h). However, addition of Dox 24 h after RCF + Ixz decreased cell death after another 24 h. **(C)** Addition of Dox (+) to induce knockdown of CypA increased Ixz (72 h) and RCF + Ixz (27 h) cell death in LNCaP/shCypA-1 and -2. **(D)** Addition of Dox (+) to induce knockdown of CypB increased cell death in Ixz but not in RCF + Ixz treated LNCaP/shCypB-1 and -2. In all cases, addition of Dox to LNCaP/shScr negative control cells did not result in differences in cell death. In addition, no Dox (−) cell death was similar to negative control cells. In the shorter RCF + Ixz (27 h) treatments, cells were pretreated with Dox for 48 h. *p* values are shown above the bars.

Figure S13

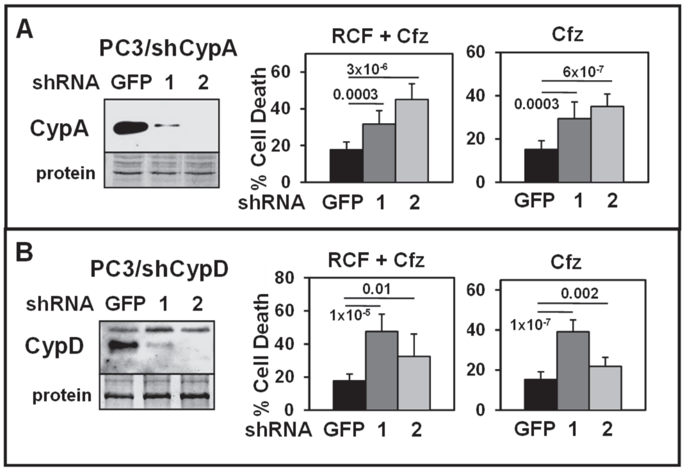

Figure S13. Stable knockdowns: CypA and CypD are pro-survival in RCF + Ixz treated PC3 CRPC cells. Westen blot analysis of stable lentivirus knockdown **(A)** PC3/shCypA-1, -2 and **(B)** PC3/shCypD-1, -2 showed decreased CypA and CypD compared to negative control PC3/shGFP cells. Protein refers to Coomassie blue stain after all immunological analysis was completed. Trypan blue exclusion assays showed treatment with RCF (1 μM) + Cfz (25 nM) (48 h) or Cfz alone (72 h) increased cell death in **(A)** PC3/shCypA-1, -2 and **(B)** PC3/shCypD-1, -2 compared to PC3/shGFP. *p* values were shown near the bars.
