# Supplementary material for: Cyclophilin Inhibitor Rencofilstat Combined with Proteasome Inhibitor Ixazomib Increases Proteotoxic Cell Death in Advanced Prostate Cancer Cells with Minimal Effects on Non-Cancer Cells
Source: Biomedicines. 2025 Oct 7;13(10):2442. doi: 10.3390/biomedicines13102442 (PMC12561735; doi:10.3390/biomedicines13102442)
Supplement: Supplementary file 1 [file biomedicines-13-02442-s001.zip › Uncropped Western Blots.pdf]

Cyclophilin Inhibitor Rencofilstat Combined with Proteasome Inhibitor Ixazomib Increases Proteotoxic Cell Death in Advanced Prostate Cancer Cells with Minimal Effects on Non-Cancer Cells

Carlos Perez-Stable, Alicia de las Pozas, Medhi Wangpaichitr, Robert Foster, Daren Ure

Uncropped Western Blots: Figures 1-6

1. **Blots were cut horizontally to analyze high, medium, or low molecular weight target proteins.**
2. **Regions used in figures are contained within red rectangles.**

Figure 1B

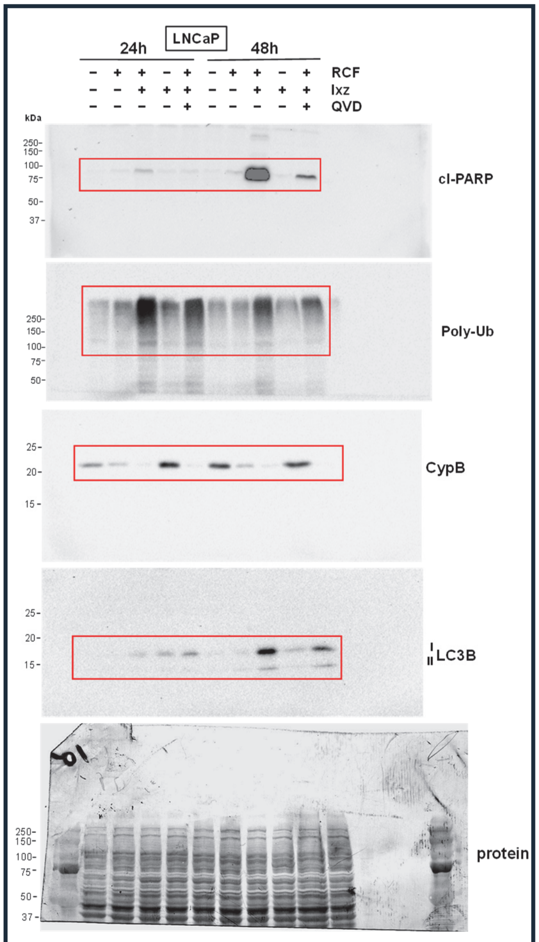

Western blot 5/11/2023

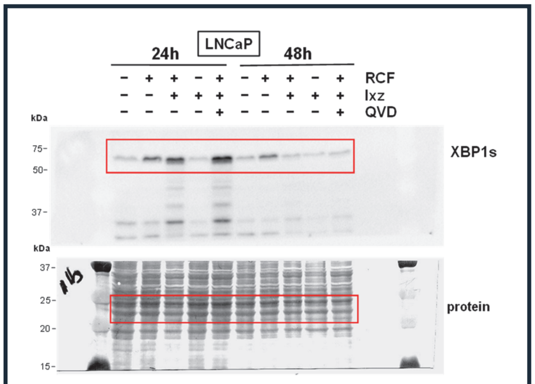

Western blot 6/7/2023

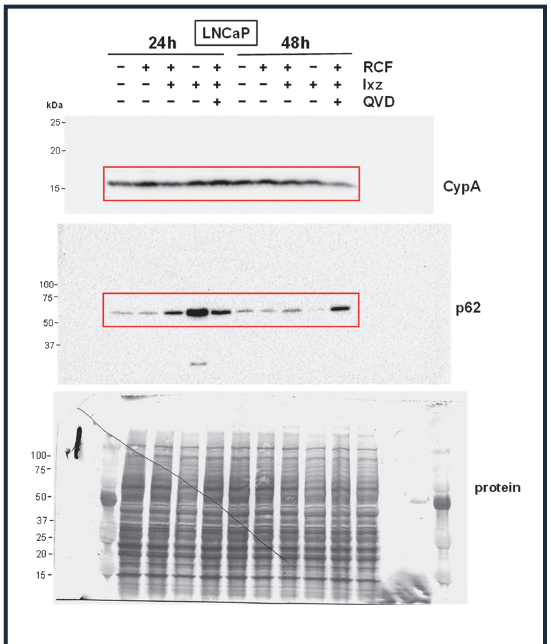

Western blot 4/19/2024

Figure 1C

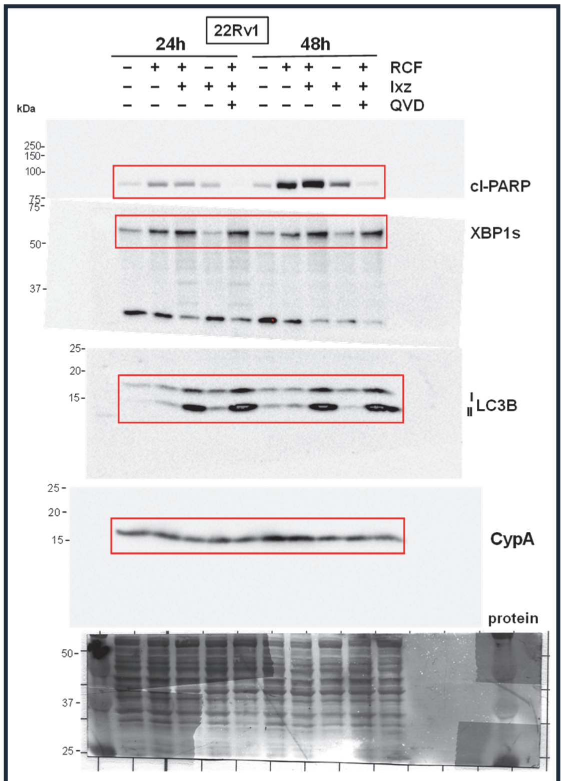

Western blot #1: 1/18/2024

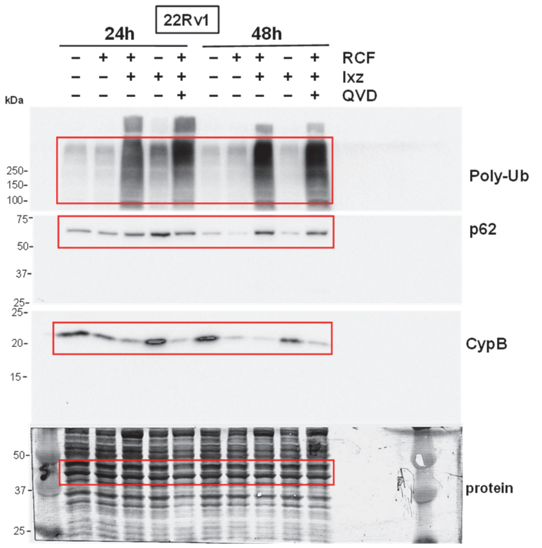

Western blot #2: 1/18/2024

Figure 1D

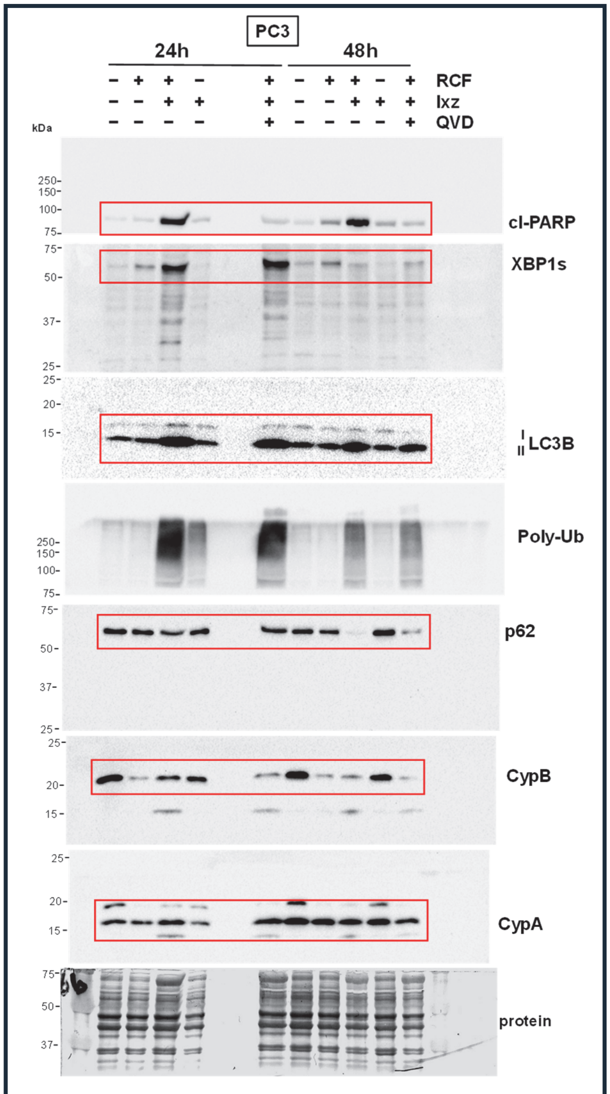

Western blot 12/13/2023

Figure 2A

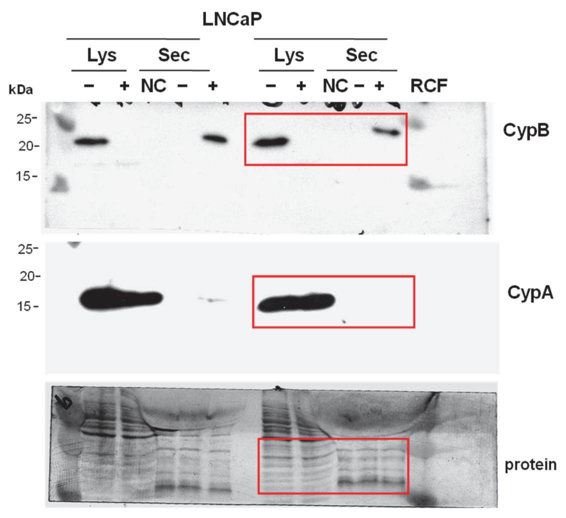

Western blot 9/28/2018

Figure 2B

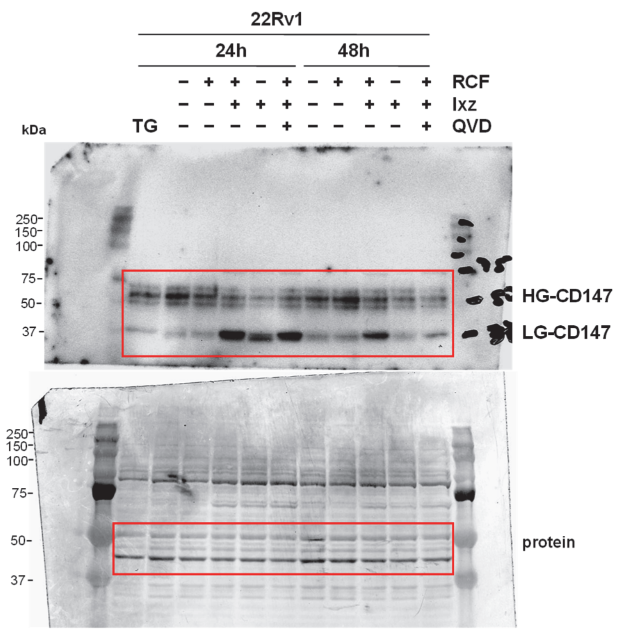

Western blot 9/24/2018

Figure 2B

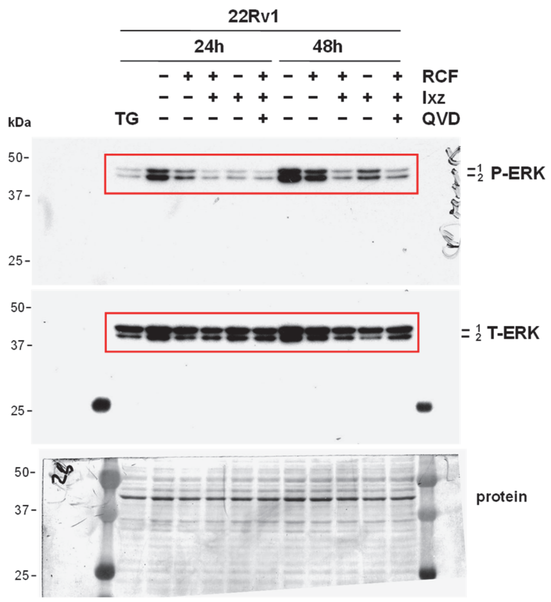

Western blot 9/26/2018

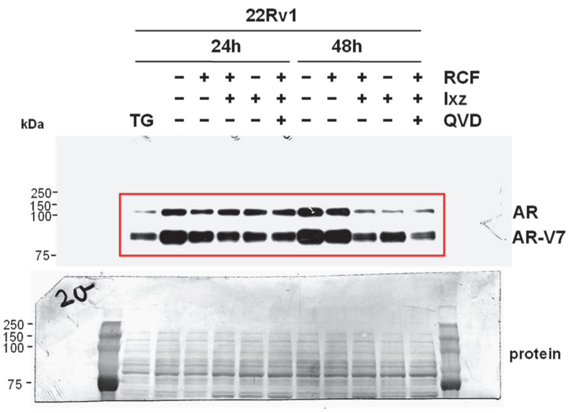

Western blot 9/26/2018

Figure 2B

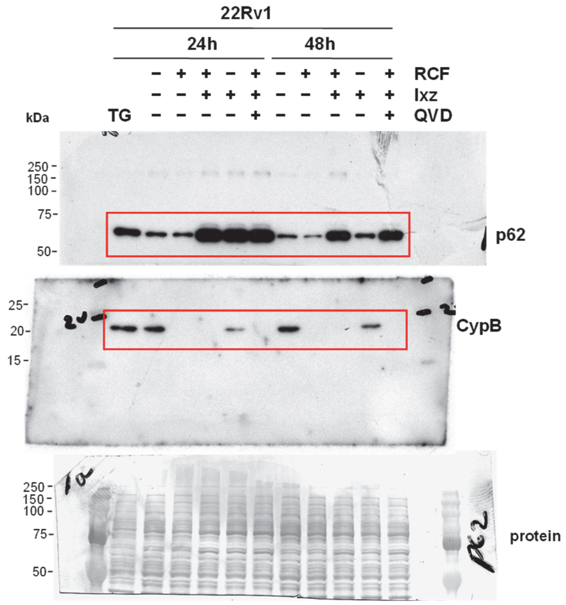

Western blot 9/27/2018

Figure 3B

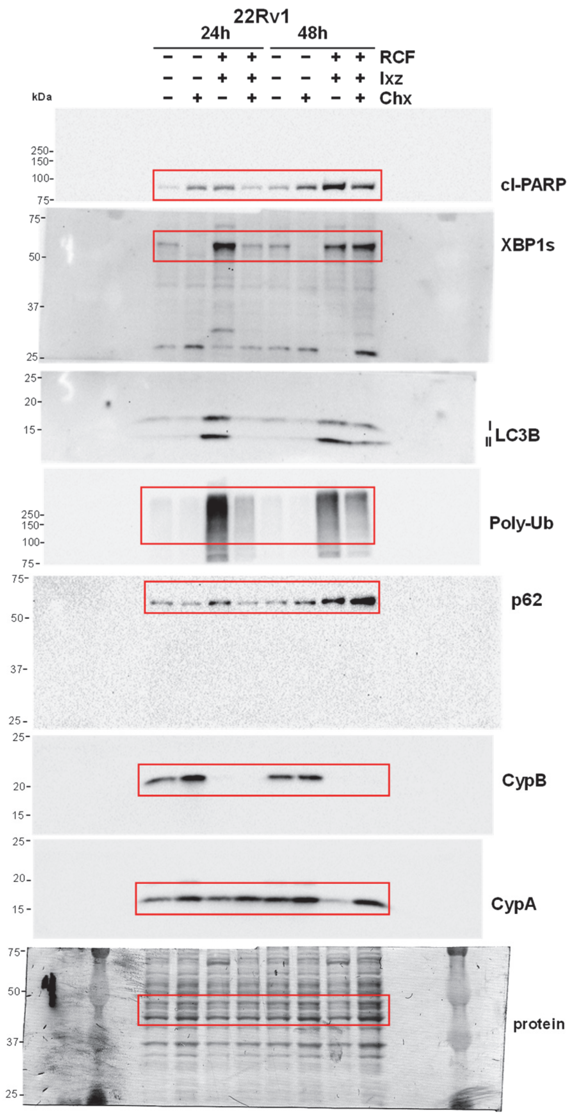

Western blot 1/26/2024

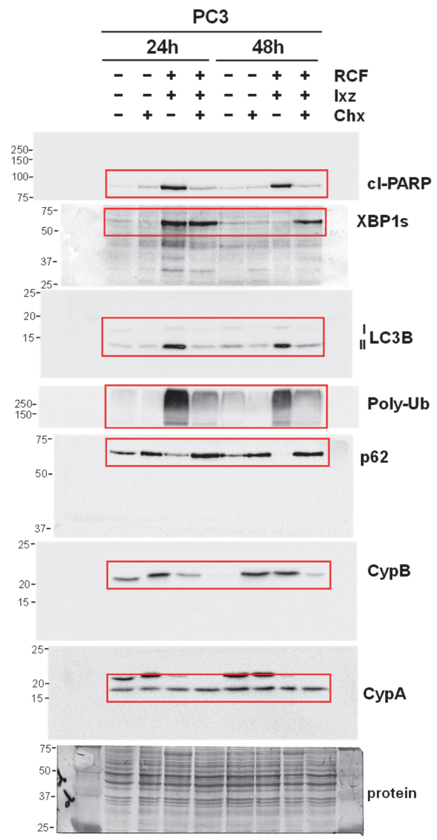

Western blot 12/21/2023

Figure 3C

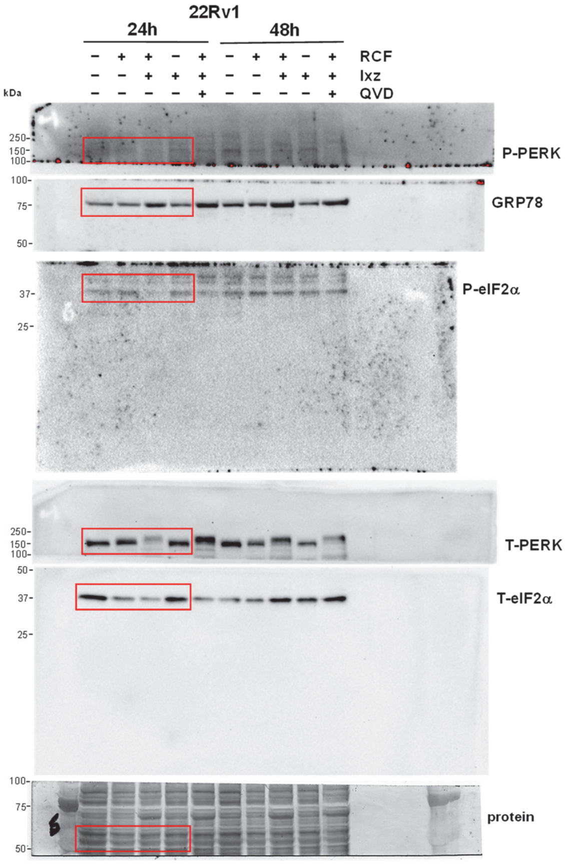

Western blot #2: 4/24/2024

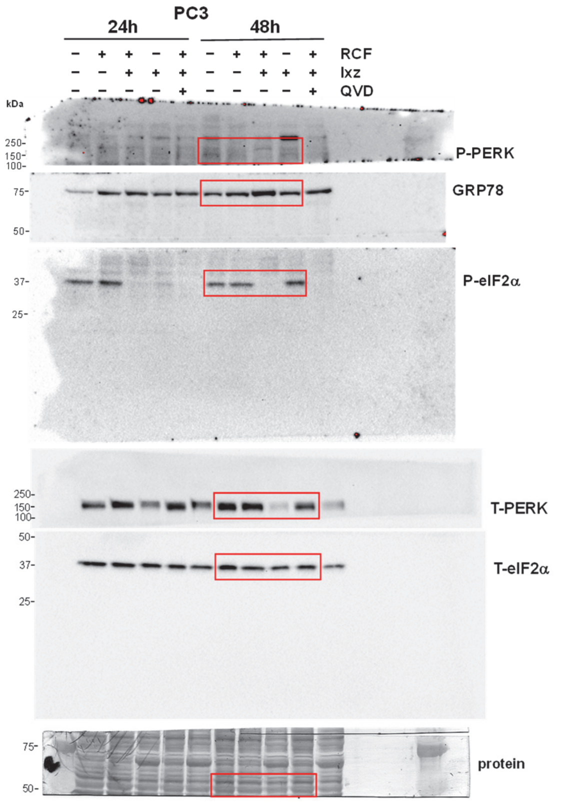

Western blot #1: 4/24/2024

Figure 4B

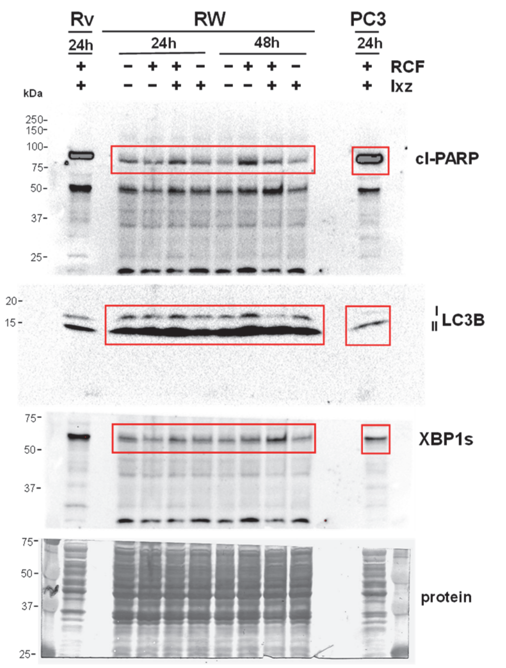

Western blot #1: 4/26/2024

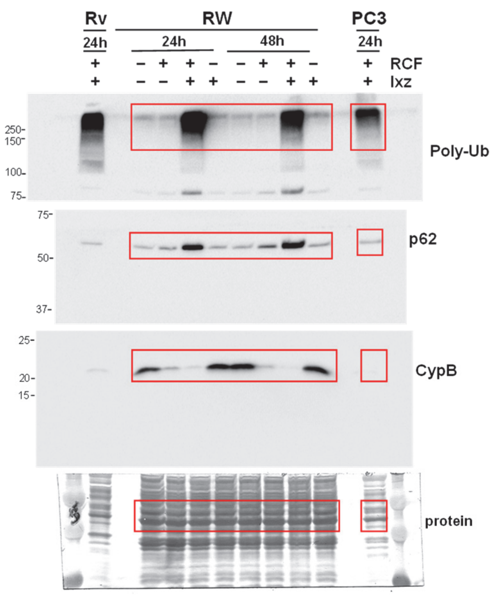

Western blot #2: 4/26/2024

Figure 4C

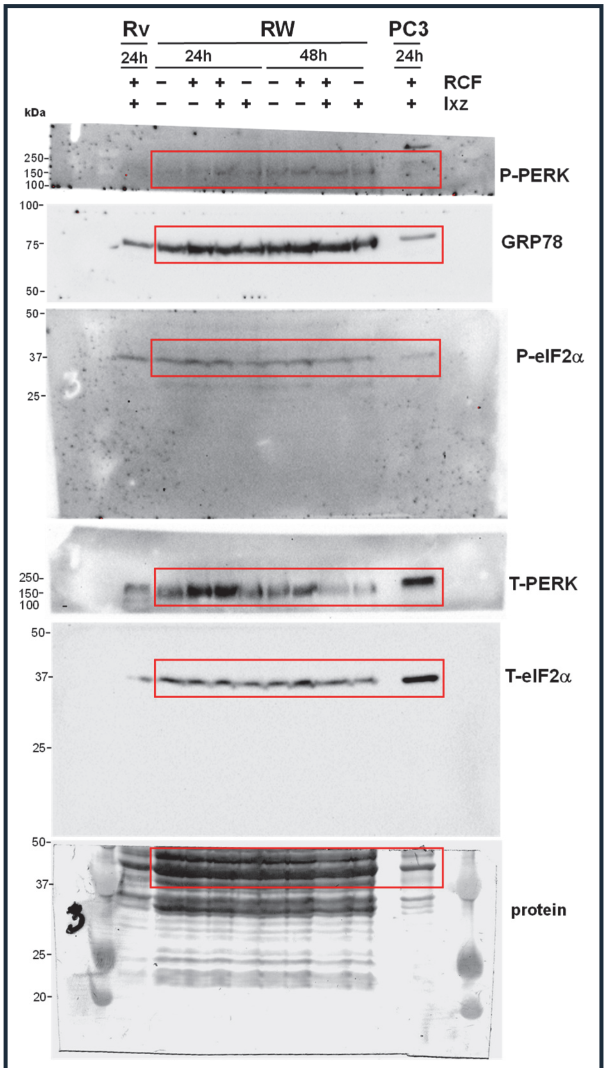

Western blot 6/13/2024

Figure 5A

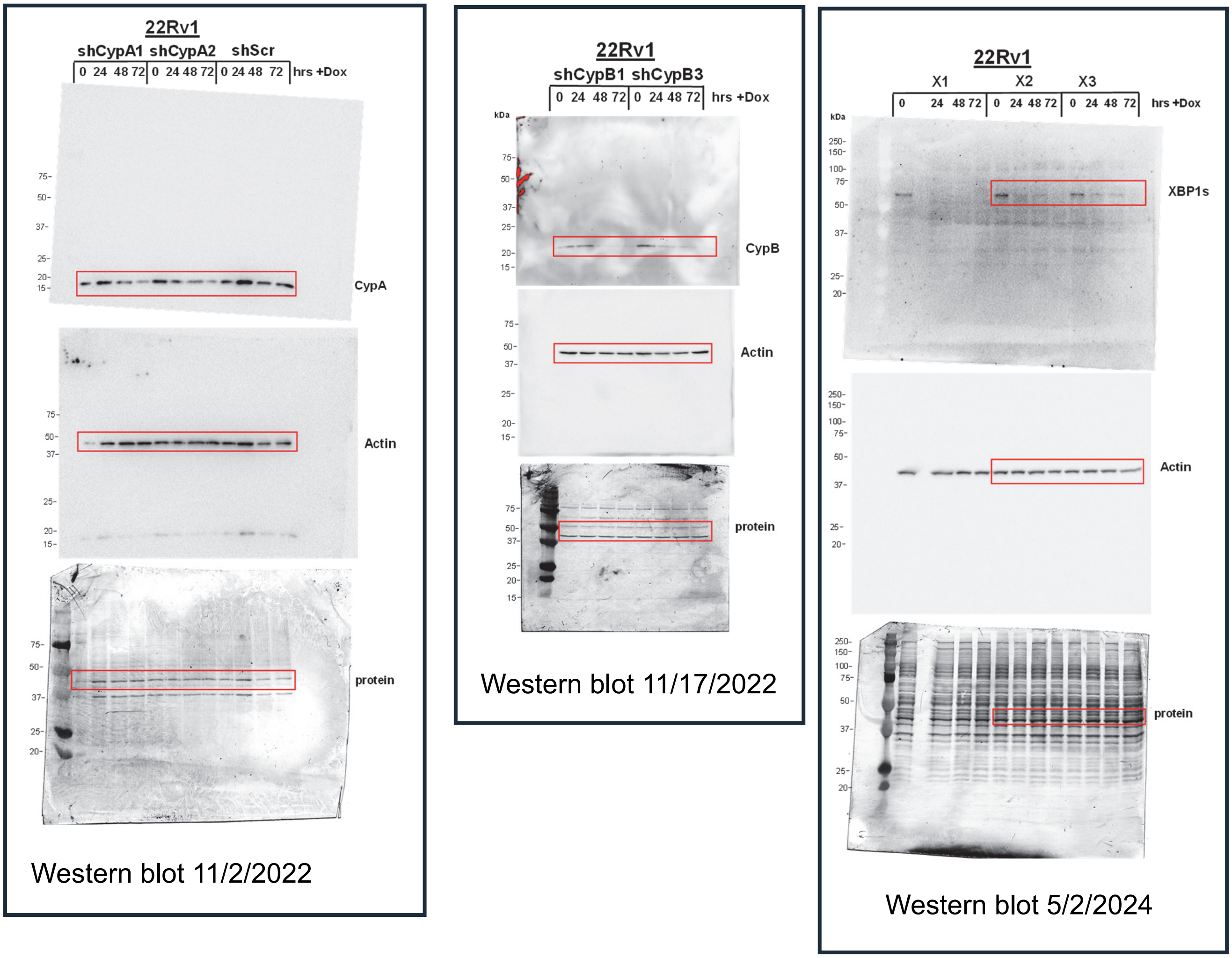
22Rv1
shCypA1 shCypA2 shScr
0 24 48 72 0 24 48 72 0 24 48 72 hrs +Dox
CypA
Actin
protein
Western blot 11/2/2022
22Rv1
shCypB1 shCypB3
0 24 48 72 0 24 48 72 hrs +Dox
kDa
CypB
Actin
protein
Western blot 11/17/2022
22Rv1
X1 X2 X3
0 24 48 72 0 24 48 72 0 24 48 72 hrs +Dox
kDa
XBP1s
Actin
protein
Western blot 5/2/2024

Figure 6A

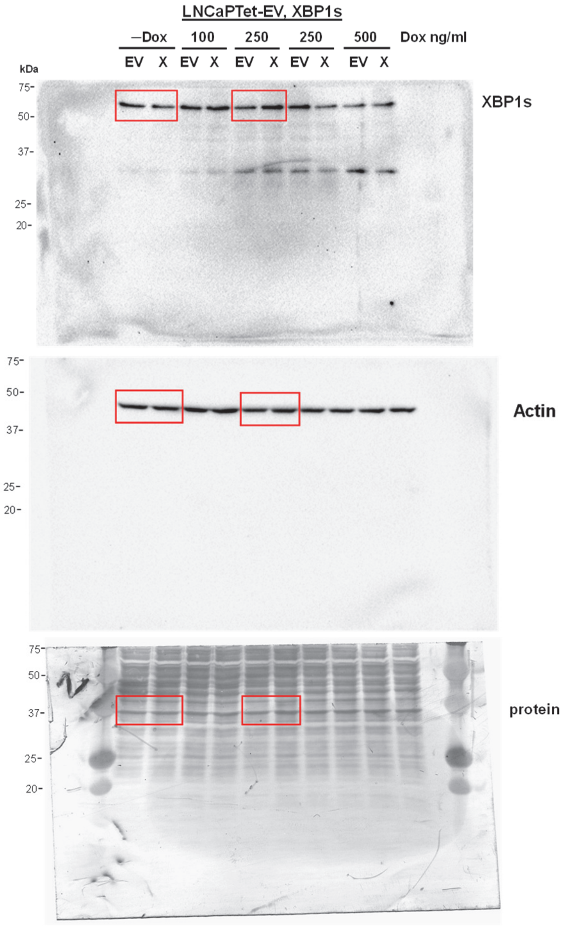

Western blot #2: 3/15/2024

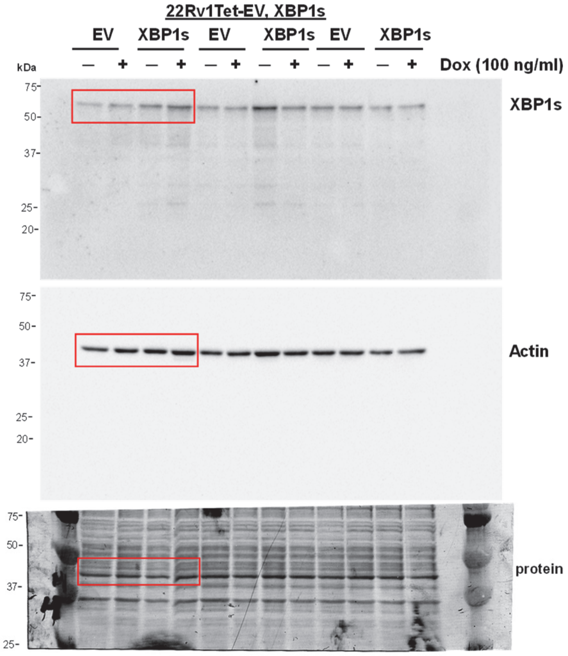

Western blot 1/23/2024

Figure 6B

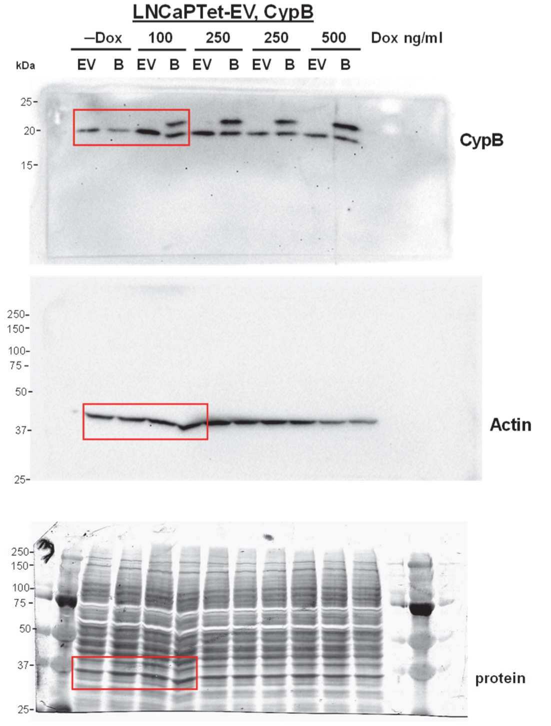

Western blot #1: 3/15/2024

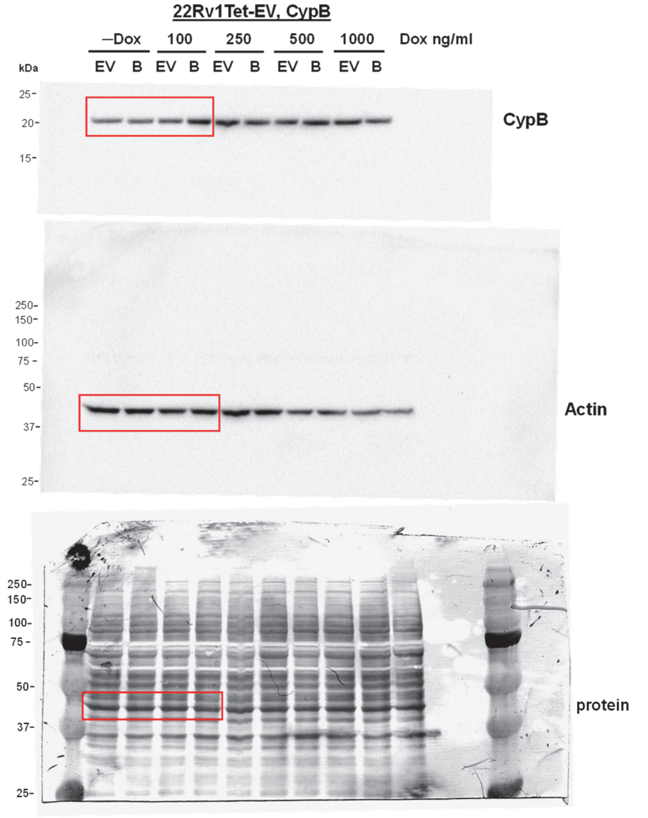

Western blot 2/9/2024

Cyclophilin Inhibitor Rencofilstat Combined with Proteasome Inhibitor Ixazomib Increases Proteotoxic Cell Death in Advanced Prostate Cancer Cells with Minimal Effects on Non-Cancer Cells

Carlos Perez-Stable, Alicia de las Pozas, Medhi Wangpaichitr, Robert Foster, Daren Ure

Uncropped Western Blots: Supplementary Figures S5-9, S11-13

1. **Blots were cut horizontally to analyze high, medium, or low molecular weight target proteins.**
2. **Regions used in figures are contained within red rectangles.**

Figure S5A

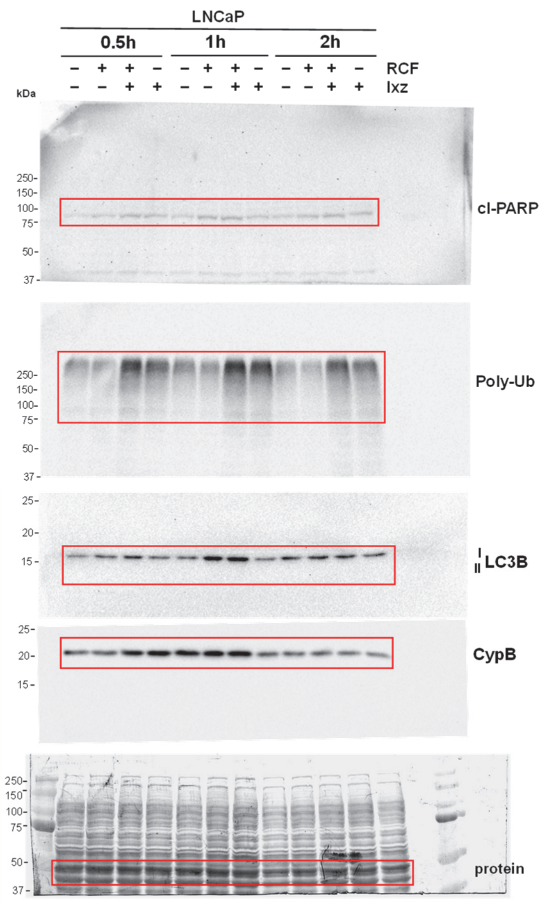

Western blot 5/11/2023

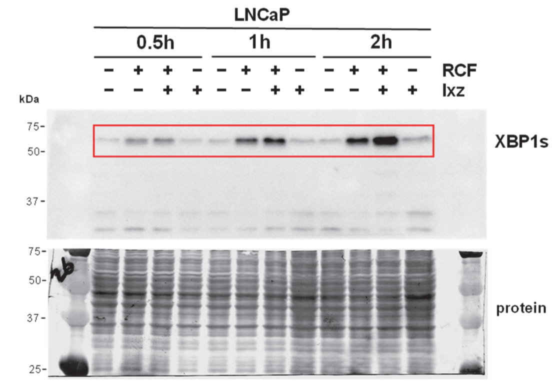

Western blot 6/7/2023

Figure S5B

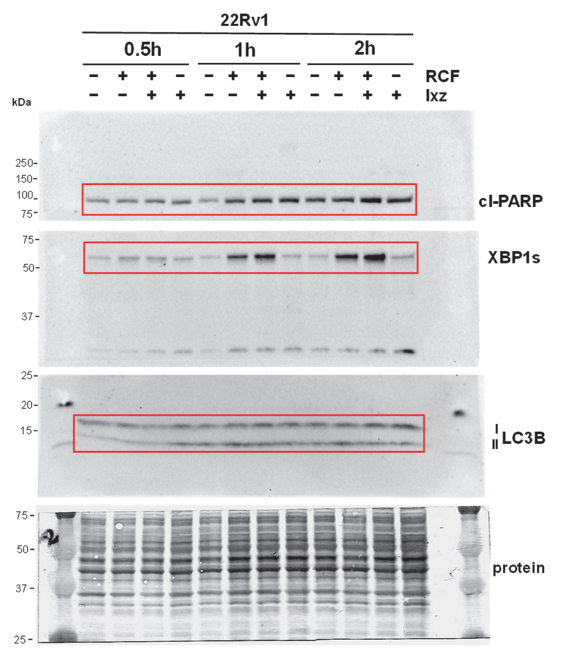

Western blot #1: 2/16/2024

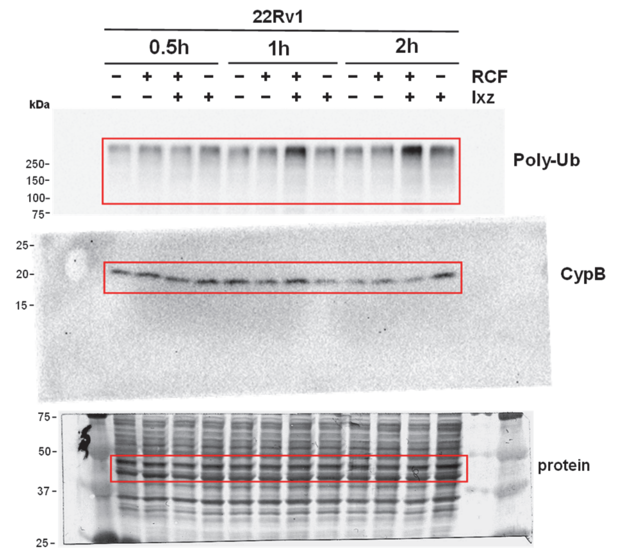

Western blot #2: 2/16/2024

Figure S5C

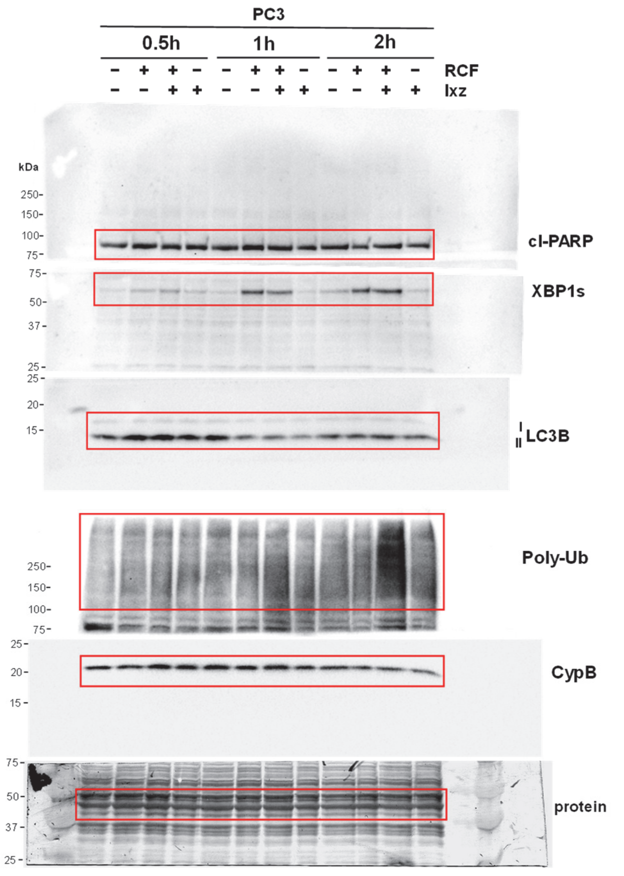

Western blot 12/29/2023

Figure S5D

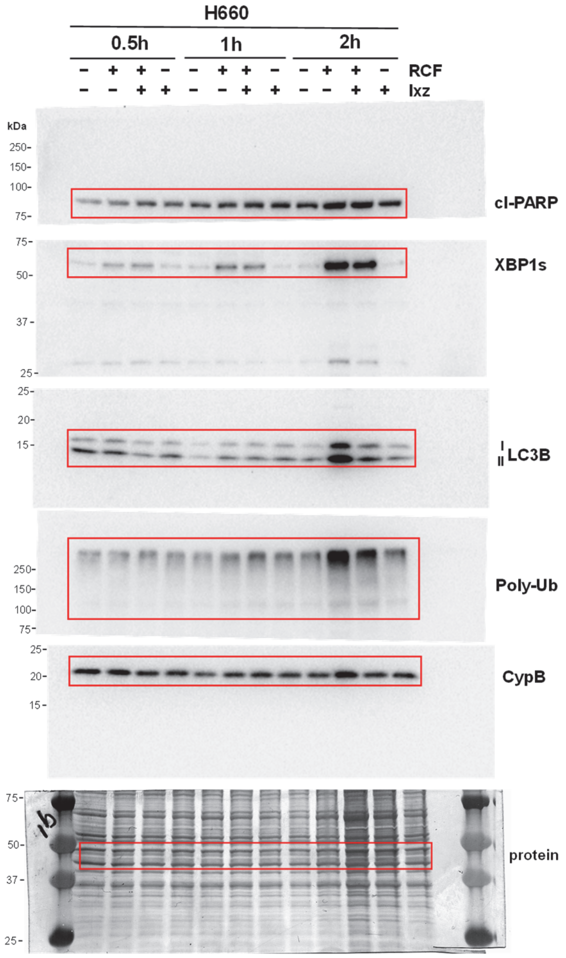

Western blot 6/28/2022

Figure S5E

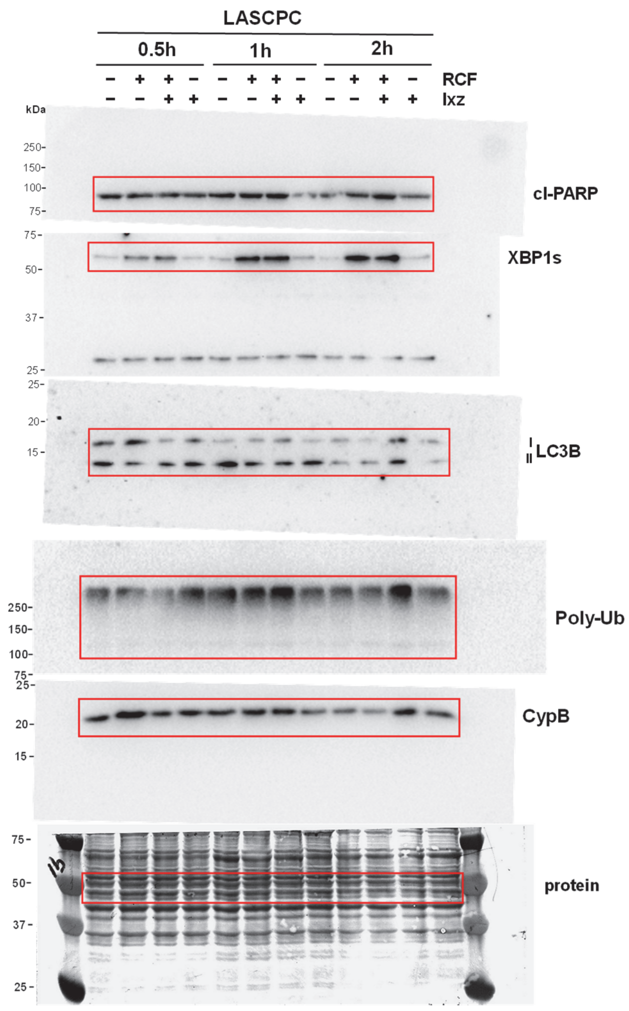

Western blot 6/23/2022

Figure S6A

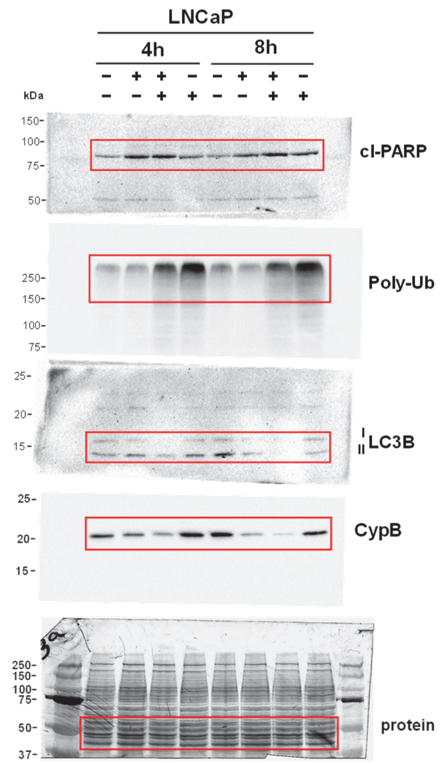

Western blot 5/12/2023

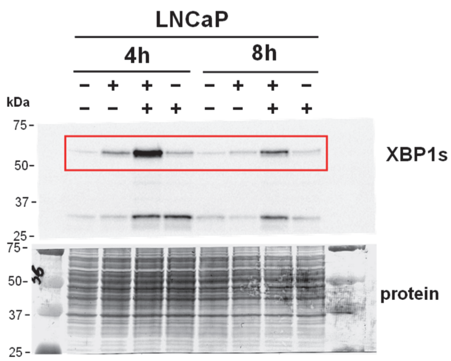

Western blot 6/9/2023

Figure S6A

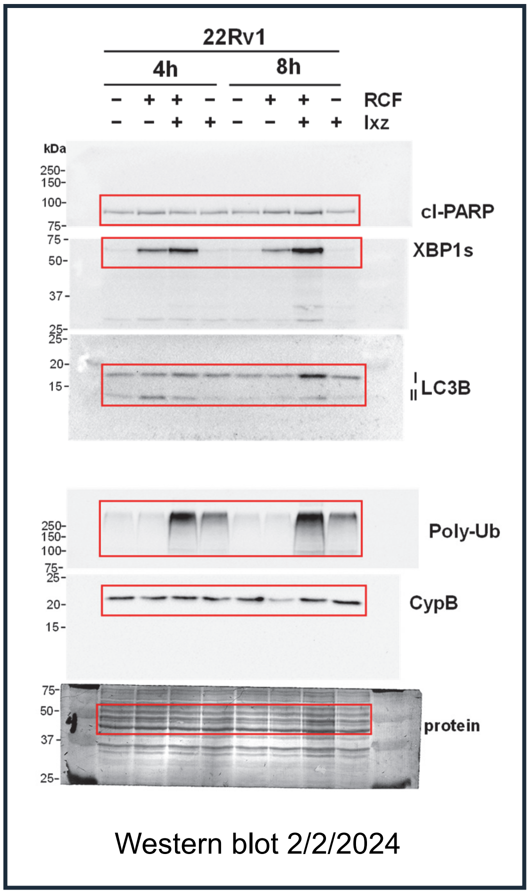

Figure S6B

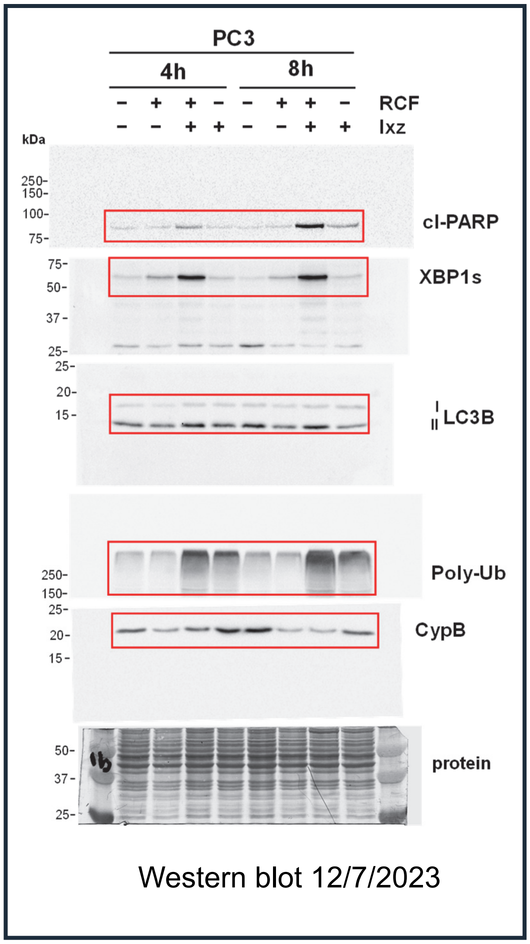

Figure S6C

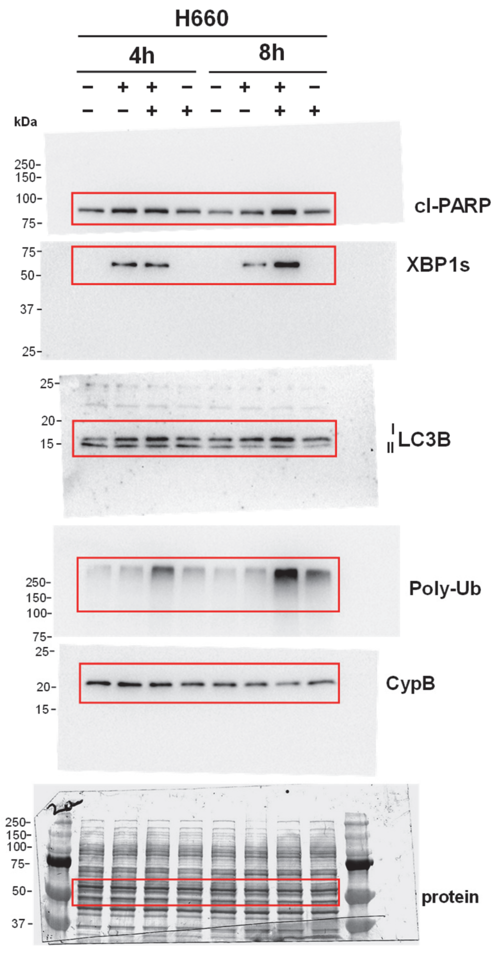

Western blot 6/17/2022

Figure S6C

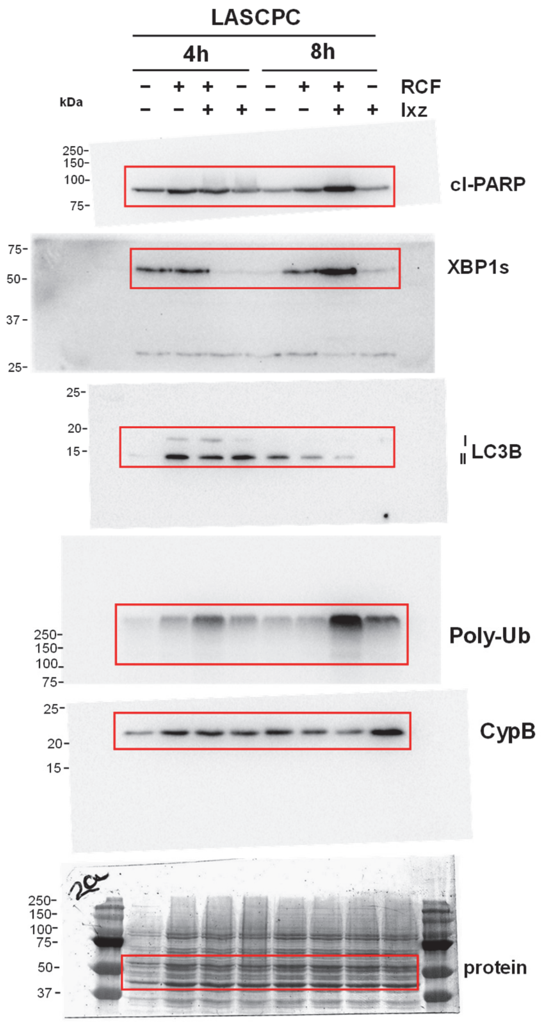

Western blot 6/14/2022

Figure S7

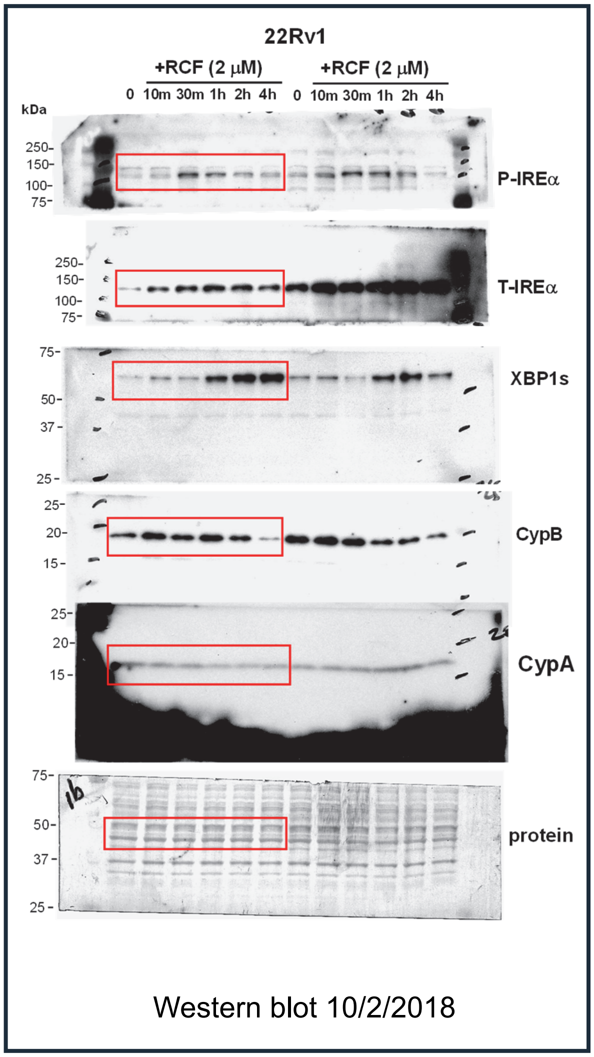
22Rv1
+RCF (2 μM)
+RCF (2 μM)
0 10m 30m 1h 2h 4h 0 10m 30m 1h 2h 4h
kDa
250-
150-
100-
75-
P-IREα
T-IREα
XBP1s
50-
37-
25-
CypB
20-
15-
CypA
protein
Western blot 10/2/2018

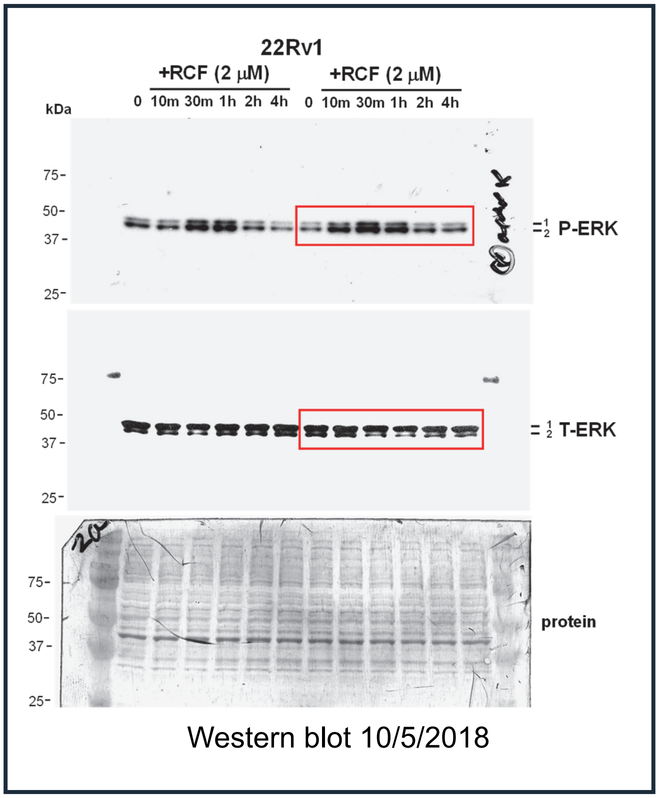
22Rv1
+RCF (2 μM)
+RCF (2 μM)
0 10m 30m 1h 2h 4h 0 10m 30m 1h 2h 4h
kDa
75-
50-
37-
25-
1
2
P-ERK
T-ERK
protein
Western blot 10/5/2018

Figure S8

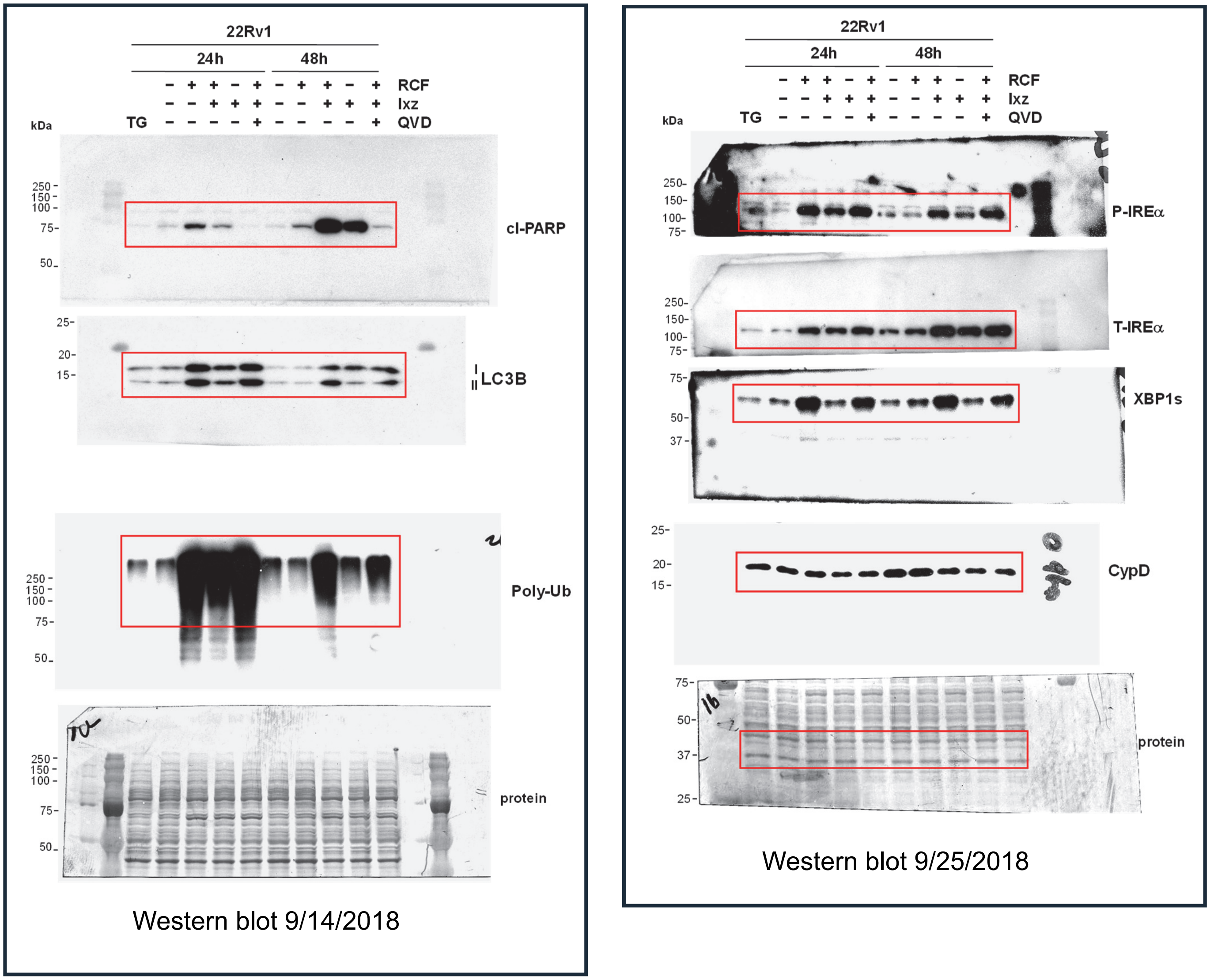
22Rv1
24h
48h
RCF
Ixz
QVD
TG
kDa
cl-PARP
LC3B
Poly-Ub
protein
Western blot 9/14/2018
P-IREα
T-IREα
XBP1s
CypD
Western blot 9/25/2018

Figure S9B

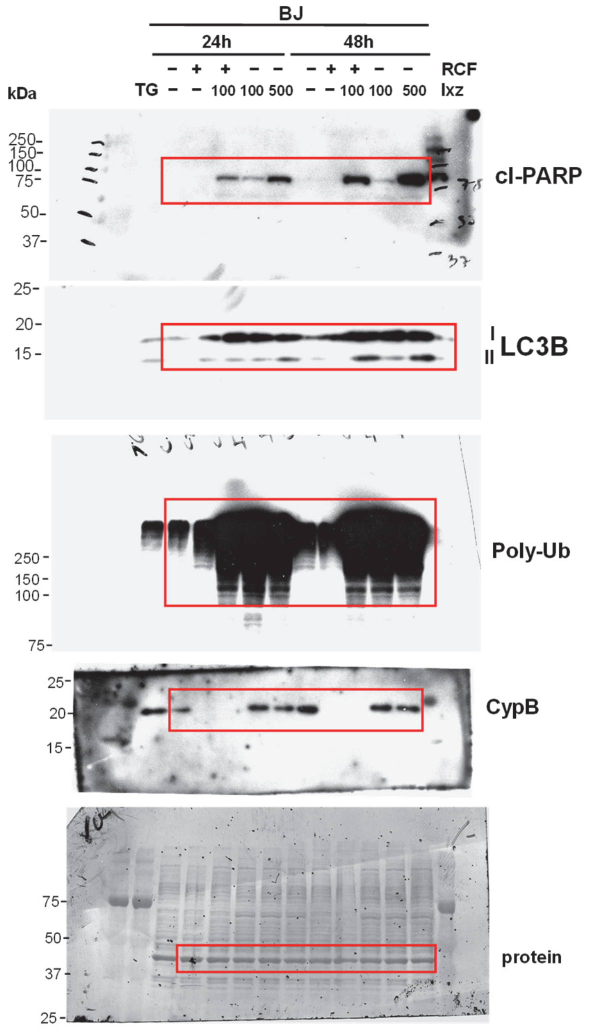

Western blot 9/20/2018

Figure S11

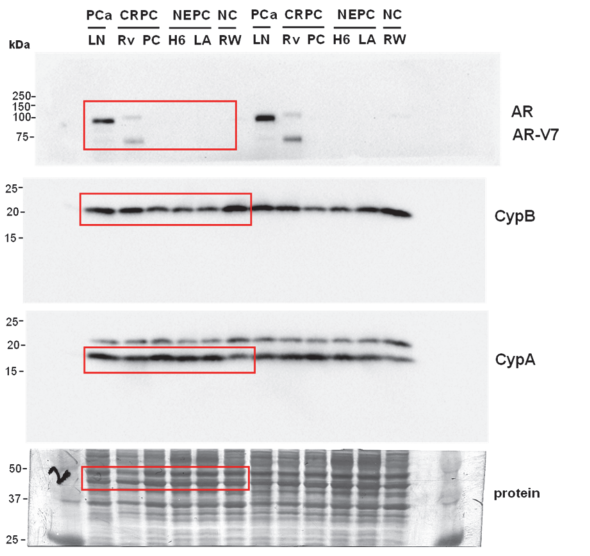

Western blot #1: 4/10/2024

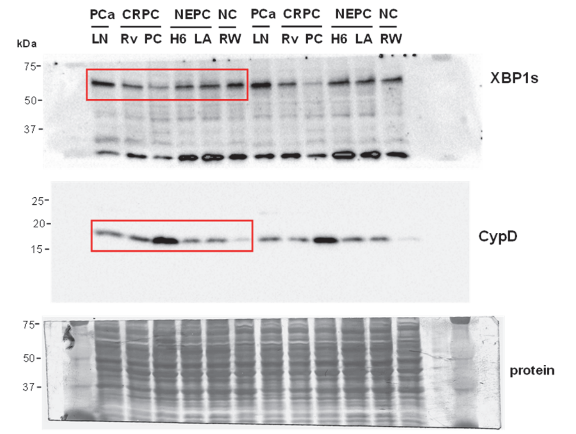

Western blot #2: 4/10/2024

Figure S12A

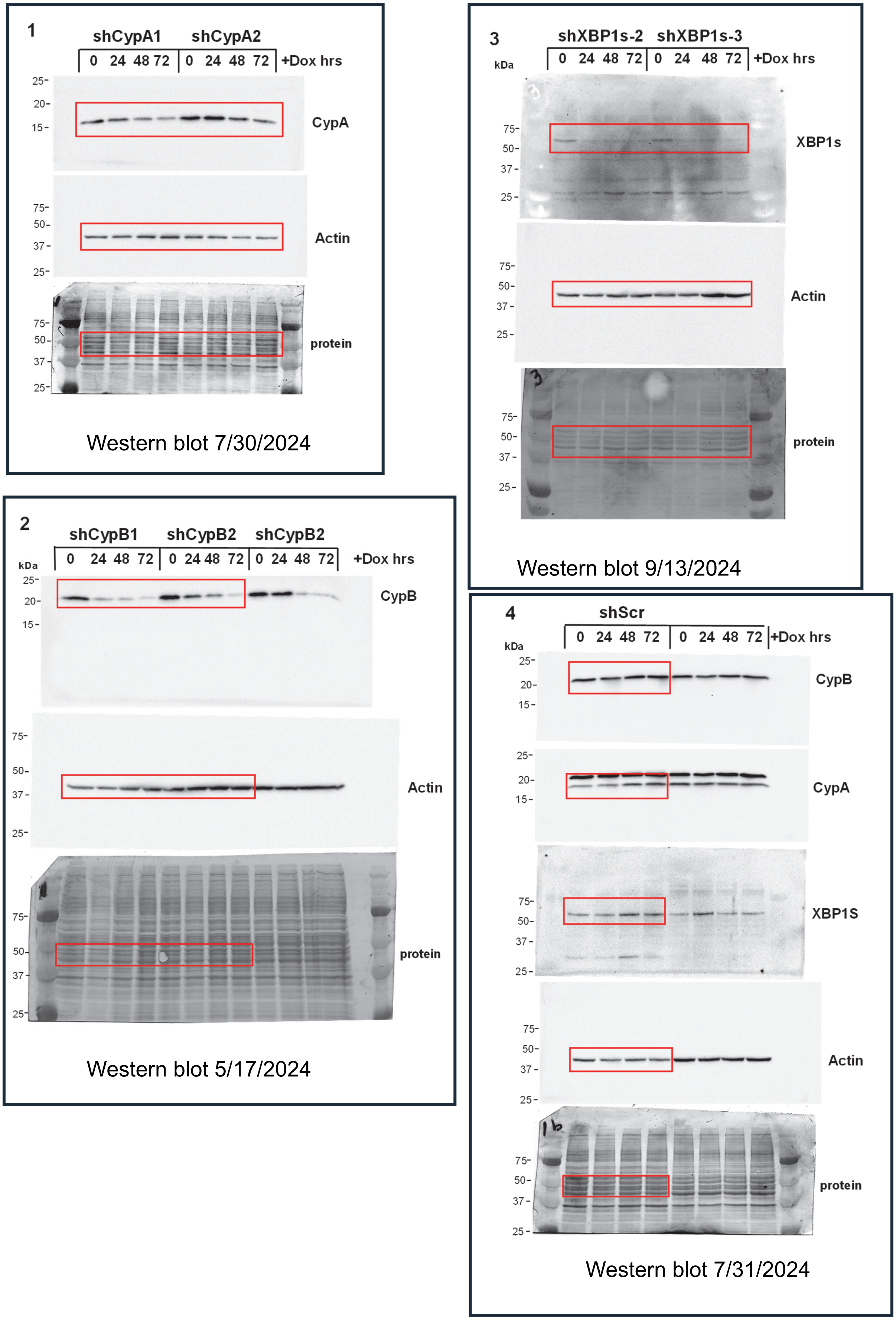
1
shCypA1 shCypA2
0 24 48 72 0 24 48 72 +Dox hrs
CypA
Actin
protein
Western blot 7/30/2024
2
shCypB1 shCypB2 shCypB2
kDa
0 24 48 72 0 24 48 72 0 24 48 72 +Dox hrs
CypB
Actin
protein
Western blot 5/17/2024
3
shXBP1s-2 shXBP1s-3
kDa
0 24 48 72 0 24 48 72 +Dox hrs
XBP1s
Actin
protein
Western blot 9/13/2024
4
shScr
kDa
0 24 48 72 0 24 48 72 +Dox hrs
CypB
CypA
XBP1S
Actin
protein
Western blot 7/31/2024

Figure S13A

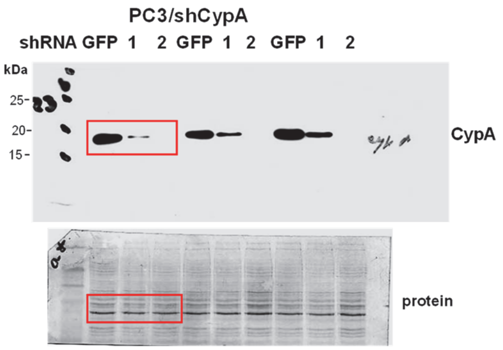

Western blot 3/4/2011

Figure S13B

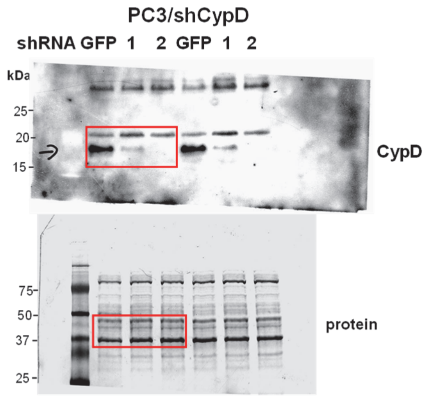

Western blot 3/30/2011
